# Progressive Medicine—Volume I, 1900

**Published:** 1901-01

**Authors:** 


Progressive Medicine—Volume I, 1900. A Quarterly Digest of Advances, Discoveries and Improvements in the Medical and Surgical Sciences. Edited by Hobart Armory Hare, M. D., Professor of Therapeutics and Materia Medica in Jefferson Medical College of Philadelphia. Octavo, handsomely bound in cloth, 404 pages, 36 engravings and a colored plate. Lea Brothers & Co., Philadelphia and New York. Issued quarterly. Price, $10.00 per year.

The plan of Progressive Medicine for last year has seemed so satisfactory, that practically no changes for this year are made. Every available source of information has been culled and the gist is given after having been weighed and thoroughly digested. This Quarterly is popular with the profession, and deservedly so. No physician, who wishes to review the whole literature four times a year can afford to be without these books. T. J. B.

